# COVID 19: are South African junior doctors prepared for critical care management outside the intensive care unit?

**DOI:** 10.11604/pamj.2021.40.41.30134

**Published:** 2021-09-16

**Authors:** Nadiya Ahmed, Ryan Davids

**Affiliations:** 1 Department of Surgical Sciences, Department Anaesthesiology and Critical Care, Tygerberg Hospital, Stellenbosch University, Western Cape, South Africa

**Keywords:** COVID-19, junior doctor, critical care, South Africa

## Abstract

**Introduction:**

the coronavirus disease 2019 (COVID-19) pandemic has negatively impacted countries across the globe. Infected individuals will seek aid at various health care facilities. Many patients will recover without requiring specialised treatment. A significant percentage of infected individuals will need critical care management, which will begin in the emergency department, generally staffed by junior doctors. Junior doctors will need to stabilize, triage and manage these patients prior to referral to specialized units. Above and beyond the usual occupational demands that accompany junior doctors in state facilities, this pandemic will thrust further responsibility on them. The objectives were to describe crisis preparedness of junior doctors in the areas of triage decision-making and critical care management, outside the intensive care unit.

**Methods:**

this is a descriptive, cross-sectional study, utilizing a web-based survey. Junior doctors in South Africa, being doctors in year one or year two of internship and community service, were invited to participate anonymously via various social media platforms. **Results:** a total of 210 junior doctors across South Africa answered the survey. Junior doctors expressed confidence with knowledge of intubation drugs, to perform intubation and cardiopulmonary arrest resuscitation without supervision. Only 13.3% of respondents expressed comfort with setting and adjusting ventilator settings independently. 57% of participants expressed discomfort with making critical care triage decisions. Ninety-three percent (93%) of participants expressed benefit from a telemedicine intervention.

**Conclusion:**

junior doctors in South Africa indicate that they are prepared to initiate management of the critically ill patient outside the intensive care unit but remain uncertain in their ability to provide ongoing critical care management. The COVID-19 pandemic has highlighted the need to prepare junior doctors with the ability to manage critical care triage and management in emergency rooms. Leveraging of the workforce in South Africa may be potentiated by telemedicine interventions.

## Introduction

The South African health care system relies heavily on the fraternity´s newest recruits [1]. The interns and community service doctors execute an immense clinical and administrative function within the South African public health care sector. This sector caters to the needs of more than 59 million people. More than 80% of this population is serviced by the public health care sector, which has one doctor to patient ratio of 26 per 100 000 [2]. A hardship in the South African public health care sector is the limited access South Africans have to intensive care. International norms show an eight-fold indifference in intensive care unit (ICU) bed availability across the developed countries. In 2012, this was documented as many as 25 beds per 100 000 population in the United States of America [3]. The total number of critical care beds last reported (2009) in South Africa is 4 719 across all sectors with a 1: 10 000 bed to patient ratio, recognising that this is not an equal distribution between provinces, urban and rural areas [4]. In the face of the SARS-CoV-2 pandemic the above numbers highlight the weight of limited access to critical care beds for South Africans, with similar sentiments echoed throughout Africa and the world. At one point, the projected disease burden was approximately 20 - 40% of SA´s population would be infected with the virus. In essence, it predicted 500 000 to 1 million infected, the majority of whom would need medical attention and hospitalization. Based on global trends a minimum of 5% of the infected will need critical care, equating to approximately 25 000 - 50 000 patients with need of critical care [5]. The health care sectors worldwide, and more so in the developing world, are not equipped to deal with these numbers nor do they have the required numbers of skilled manpower and equipment needed to present a response to meet the demand of these projected numbers. The burden thus falls on the expertise and resources at our nation´s disposal and relies on the rapid equipping of personnel to meet the expected inundation [6]. What we have learnt thus far, is the need for every medical doctor, irrespective of specialisation, avail themselves to frontline response in being a generalist doctor and provide the initiation of critical care management outside the intensive care unit. The ability to diagnose respiratory distress, adequately manage organ dysfunction, offer palliative care and often end of life care is key in crisis situations [7]. In the evolution of medical specialisations this was the task of the pulmonologist, intensivist and palliative care specialist respectively. As the profession of medical doctors progressed into further organ specialisation, the less we became attuned to being generalists [8].

The generalists at this stage remain the intern doctors, community service medical officers and medical officers. This has led to question our teaching in the undergraduate programmes as well as during the internship process of 2 year service in a public hospital to work under supervision so as to hone much needed integration of knowledge and clinical skills required for critical care management [9]. Timely recognition of an acutely deteriorating patient is paramount to survival [7, 10]. When compared to the specialties of Internal Medicine, Surgical Sciences and Anesthesiology, critical care is relatively new in our country [11, 12]. For those who are not specialised in critical care, in particular doctors at the interface of undergraduate and postgraduate training, the ability to manage critical patients is reliant on self-integrated skill and knowledge drawn from the disciplines of internal medicine, anaesthesiology and general surgery [13]. Intensive care physicians are still coming to terms with the clinical difficulties in managing this pandemic let alone the emotional burden and burn out associated with end of life decisions and care [11, 14]. Medical ethics and rationalising resource limitations to best prognostic cases has not always featured in the medical undergraduate curriculum in South Africa [15]. Evidence has shown the invaluable benefit that aforementioned has to junior doctors across the globe [16, 17]. Despite this, it has not been incorporated nationally into undergraduate training programmes. It has been reported that junior doctors of the Mandela Castro programme who served to bolster the public health care service, felt ill-equipped to manage the clinical landscape in South Africa, and uncertainty prevails around how well they would cope with the palliative and end of life care decisions as well as the value of a telemedicine platform for these difficult clinical scenarios [18]. Much has been written about the resource allocation and the necessary equipment needed to fight the COVID pandemic, but little is offered in line of human resources, particularly the readiness of the frontline doctors [18]. The authors aim to identify the deficits in clinical management and ethical decision-making by the junior doctor in managing critically ill patients outside the intensive care unit.

## Methods

**Study design:** this is a descriptive cross-sectional study which utilizes a web-based survey. It was widely distributed to junior doctors. Junior doctors for purposes of this study included first and second year interns and community service doctors throughout South Africa. Medical officers in the public health sector and general practitioners in the private health care setting were excluded.

**Settings:** the inclusion criteria for this study was clearly indicated on the invitation to participate. The total number of employed interns and community service doctors is not known in South Africa, thus making it difficult to establish a sample size. The survey was repeatedly distributed and advertised over a period of six months (May 2020 to October 2020) to achieve best yield. The distribution tools included various forms of social media.

**Participants:** participants were incentivised with three lucky draw vouchers. Ethical approval was gained from Stellenbosch University Health and Research Council [USHREC1-2020-15120].

### Survey design

The survey instrument was designed, highlighting conceptual areas around critical care triage decision-making and management of the patient requiring critical care outside of the ICU. A total of 15 questions were asked, with 11 questions measured on a 4-point Likert scale. One question pertained to cardiopulmonary resuscitation, two questions to intubation, three questions to arterial blood gas analysis and ventilation. Three questions pertained to the level of comfort to counsel family of a critically ill patient, family of a dying patient and to support health care workers around a dying patient or patient that has died. One question asked about the comfort with refusing critical care management to a patient. Two questions were asked on the benefits of a South African tailored telemedicine platform. The question types varied between yes/no answers as well as Likert scale analyses ranging to assessing comfortability with specific tasks. The survey tool underwent face-validation, reviewed by two intensivists, two pulmonologists and two anaesthesiologists in the field, revised based on their recommendations.

**Data analysis:** the data was captured on an Excel spreadsheet and analysed using SPSS version 21. Data was analysed as means and medians.

## Results

A total of 210 junior doctors across South Africa completed the web-based survey. Participants were categorized according to year of mandatory public health service in South Africa, namely internship year 1 (INTERN Y1), internship year 2 (INTERN Y2) and community service medical officers (CSO). The participant breakdown was 34% (n = 71) from internship year 1, 42% (n = 88) internship year 2 and 24% (n = 50) community service medical officers. One participant did not disclose their current year of employment. On the questions pertaining to intubation and peri-intubation management, the majority of junior doctors (Mean = 58.3) self-reported knowledge of the choice of emergency intubation drugs. Under supervision, the majority of respondents reported comfort with the ability to perform intubation (Mean = 22.3), including a peri-intubation arrest and cardio-pulmonary resuscitation (Mean = 28.7). Similarly, the bulk of respondents were less confident to initiate ventilator settings (Mean = 31) and adjust ventilator settings according to patient response (Mean = 32) without supervision (Table 1).

**Table 1:** variables pertaining to intubation and peri-intubation management

Variable	Level of training			Statistical variables	
	Internship Year 1- n (%)	Internship Year 2 - n (%)	Community service medical officer - n (%)	Mean (X)	Median
**Knowledge of medications to use for an emergency intubation**					
No	22 (31%)	8 (10%)	5 (10%)	11.7	8
Yes	49 (69%)	81 (90%)	45 (90%)	58.3	49
**Comfort with intubation**					
Comfortable intubating all airways	2 (3%)	24 (27%)	22 (44%)	16	22
Only in patients with Mallempati <2	9 (13%)	42 (47%)	19 (38%)	19	23.3
Only with supervision	41 (58%)	19 (21%)	7(14%)	22.3	19
Uncomfortable with intubations	19 (27%)	4(5%)	2(4%)	8.3	9
**Comfort with running peri-intubation and cardio-pulmonary arrest**					
Not comfortable/unsure	42 (59%)	33 (37%)	8 (16%)	27.7	32
Yes, with supervision	19 (27%)	47 (53%)	20 (40%)	28.7	20
Yes, without supervision	10 (14%)	9 (10%)	22 (44%)	13.7	10
**Comfort with setting up a ventilator**					
Not comfortable/unsure	49(69%)	29 (33%)	15 (30%)	31	29
Yes, with supervision	20 (28%)	43 (48%)	14 (28%)	25.7	20
Yes, without supervision	2 (3%)	17 (19%)	21 (42%)	13.3	17
**Comfort with interpreting arterial blood gasses**					
Not comfortable/unsure	6 (8%)	5(6%)	1(2%)	4	5
Yes, with supervision	17 (24%)	18 (20%)	12 (24%)	15.7	16
Yes, without supervision	48 (68%)	66 (74%)	37 (74%)	50.3	48
**Using the blood gas to adjust the ventilator to correct abnormalities**					
Not comfortable/unsure	50 (70%)	31 (35%)	15 (30%)	32	31
Yes, with supervision	16 (23%)	41 (46%)	15(30%)	24	16
Yes, without supervision	5 (7%)	17 (19%)	20 (40%)	14	17
**Total respondents per level of training (%)**	71 (100%)	89 (100%)	50 (100%)		

**Figure 1:**
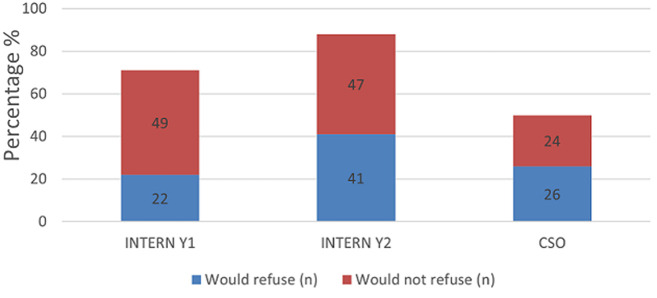
junior doctors´ comfort with critical care triage

**Figure 2:**
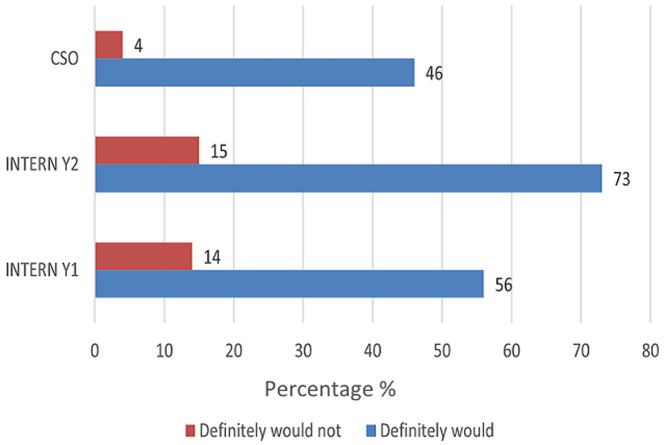
comfort with counselling family of an ill patient

**Figure 3:**
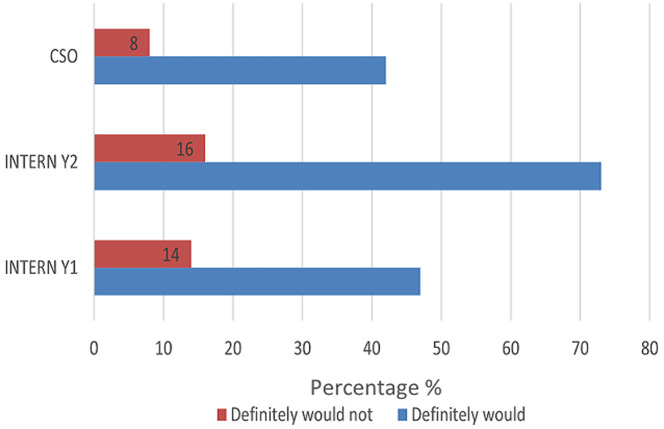
comfort with counselling family of a dying patient

**Figure 4:**
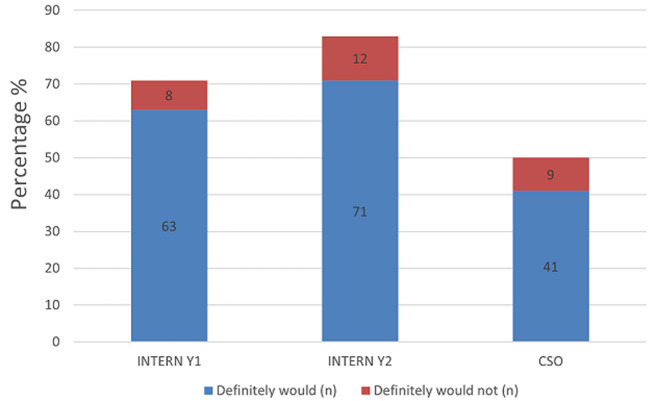
counselling of health care worker around patient death

**Figure 5:**
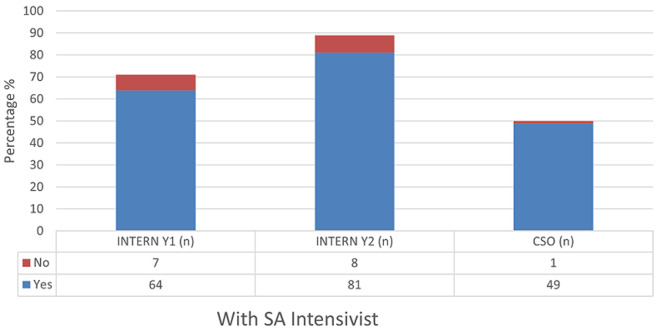
telemedicine intervention with South African intensivist

**Figure 6:**
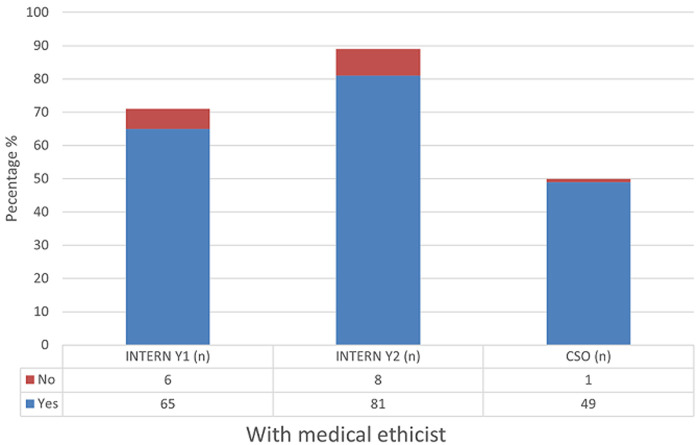
telemedicine intervention with medical ethicist

The majority of respondents self-assessed that they were able to independently interpret arterial blood gases (Mean = 50.3) (Table 1). Further analysis between each group revealed increasing self-confidence in the ability to manage intubation and peri-intubation management, with increasing years of seniority (Table 2). In each category, more than half of the participant population (57%) expressed difficulty with critical care triage, in particular the inability to refuse a patient for critical care admission (Figure 1). The preponderance of participants indicated that they felt comfortable with counselling of families of ill or dying patients (83% and 77% respectively). They also expressed comfort with providing counselling to healthcare workers around the death of a patient (83%) (Figure 2, Figure 3, Figure 4). Future telemedicine intervention with a South African qualified intensive care specialist and ethicist is viewed favorably by the majority of respondents (Figure 5, Figure 6).

**Table 2:** variables pertaining to intubation and peri-intubation management - analysis within groups

Variable	Level of training		
	Internship Year 1- n (X)	Internship Year 2 - n (X)	Community service medical officer - n (X)
**Knowledge of medications to use for an emergency intubation**			
No	22 (31)	8 (10)	5(10)
Yes	49 (69)	81 (90)	45 (90)
**Comfort with intubation**			
Comfortable intubating all airways	2 (3)	24 (27)	22 (44)
Only in patients with Mallempati <2	9 (13)	42 (47)	19 (38)
Only with supervision	41 (58)	19 (21)	7(14)
Uncomfortable with intubations	19 (27)	4(5)	2(4)
**Comfort with running peri-intubation and cardio-pulmonary arrest**			
Not comfortable/unsure	42 (59)	33 (37)	8 (16)
Yes, with supervision	19 (27)	47 (53)	20 (40)
Yes, without supervision	10 (14)	9 (10)	22 (44)
**Comfort with setting up a ventilator**			
Not comfortable/unsure	49(69)	29 (33)	15 (30)
Yes, with supervision	20 (28)	43 (48)	14 (28)
Yes, without supervision	2 (3)	17 (19)	21 (42)
**Comfort with interpreting arterial blood gasses**			
Not comfortable/unsure	6 (8)	5(6)	1(2)
Yes, with supervision	17 (24)	18 (20)	12 (24)
Yes, without supervision	48 (68)	66 (74)	37 (74)
**Using the blood gas to adjust the ventilator to correct abnormalities**			
Not comfortable/unsure	50 (70)	31 (35)	15 (30)
Yes, with supervision	16 (23)	41 (46)	15(30)
Yes, without supervision	5 (7)	17 (19)	20 (40)

## Discussion

South African intensive care units are constantly under strain. Prior to the COVID-19 pandemic, waiting lists for ICU admission were the daily norm, even after applying relatively stringent triage criteria [19]. The SARS-CoV-2 pandemic overwhelmed critical care resources in the developed world and was projected to further strain the health care sector in South Africa [5]. In view of this, all doctors had to prepare themselves to manage patients, including critically ill patients outside of the intensive care unit. Emergency units, theatres and wards had to be converted into makeshift “intensive care units”. There is a paucity of literature investigating the level of competence experienced by junior doctors in managing these patients. A previous study by van Deventer *et al*. suggested self-reported competency with intubation, with the majority of respondents feeling comfortable enough with the procedure that they could teach or supervise others. However, the study by van Deventer *et al*. did not delineate if seniority in years of practice contributed to the competency [20]. The study done here demonstrated that the self-reported comfort and competency with intubation and cardiopulmonary resuscitation exists, and is improved with increasing seniority. Intubation, arterial blood gas analysis and cardiopulmonary resuscitation are taught in the undergraduate curriculum repeatedly through the core disciplines of anaesthesiology, internal medicine, emergency medicine and surgical sciences. These skills are then strengthened with experiential learning that occurs in the initial years under the guide of senior staff. Further, junior staff is encouraged to participate in national courses that contribute to training of resuscitation such as Advanced Cardiovascular Life Support (ACLS), Advanced trauma life support (ATLS), Pediatric Advanced Life Support (PALS). The self-reported comfort in these areas can therefore be easily explained. Providing care to a critically ill patient is a step beyond resuscitation and requires the application of principles from basic physiology, pathology, internal medicine and anaesthesiology. Without a module that helps integrate these for the undergraduate medical student, the onus is on the newly qualified medical doctor to draw the golden thread that connects these when applying mechanical ventilation to a patient [13]. Ventilation is further complicated by a multitude of modes and manufacturer specific terminology [21]. This was iterated in the findings of this study, which showed that whilst most participants were comfortable with the initiation of critical care management, they reported being less confident and comfortable on how to continue to manage the ongoing ventilation that these patients require.

Across the categories, the greater number of participants reported that they would undertake the act of counseling of the family of ill and dying patients as well as to provide support to colleagues. This is reassuring given the relative limited access to oxygen and critical care beds during the COVID-19 pandemic. The majority of participants however found critical care triage decision-making to be difficult. As medical doctors, we are ingrained to try to save patients at all cost. That is inherently to the personality that is attracted to a career in the medical sciences. So, denying access to a lifesaving intervention, no matter how poor the prognosis, is seemingly counterintuitive. In a resource constrained environment, it is required to bear conscience to prioritization of patients. This enables a resource to utilize by more patients and hence provides a greater service to the community as a whole [22]. When this pandemic passes, the need to equip junior doctors to manage critically ill patients in the emergency department does not end. This has been the reality before COVID and will continue thereafter. At the current rate of critical care specialists in South Africa and internationally, it will be generations after us that will reap the rewards of undergraduate and postgraduate critical care training. This leaves us in a unique and inventive age. If we want to improve our health services for the critically ill patient, then the way to do this is by leveraging the workforce [23]. On one hand, we know that clinical outreach from central training centers to peripheral and even rural facilities improves health care access [24]. On the other, we have too few critical care specialists to perform the load of clinical responsibility and outreach, which requires hours of travel [11]. To this end, the authors probed the participant’s perception of telemedicine. The results are encouraging for future research, as there was an overwhelming positive view to engaging with a South African intensivist and/or a medical ethicist using a telemedicine platform. Technology can be used to our benefit if we intend to teach and train a generation that has grown with the internet and smartphone age, a generation whose learning is successful with technology [25]. The power that technology offers us, is the investment of time in the short term to create visual and auditory learning material suited to the South African junior doctor and place it on a forum that they are familiar with [26, 27]. The long-term benefit of this is time saved on travelling, a resource that keeps on teaching and provides continued critical care support for junior doctors working in the frontline. The study surveyed participants across South Africa in the public health sector, which has a standard or requirements to be filled in each year of mandatory service [27, 28]. Conclusions between the groups can draw, however factors (age, consultant supervision and teaching) which may vary within each group that cannot be accounted for.

### Study limitations

The study aimed to describe the confidence as reported by junior doctors but could not evaluate clinical competency. The study could not evaluate a sample size given that the exact number of interns and community service medical officers is unknown. As such, the findings of this study is not generalizable to all interns and community service medical officers.

## Conclusion

The COVID-19 pandemic has forced the academic community to re-evaluate medical practices, including the need for pandemic preparedness. In the midst of a health crisis, we are profoundly reliant on our most junior health care workers to man the frontlines and leverage the system in order to provide a sustainable and life-saving service. The authors demonstrate that these junior doctors report being comfortable with the initiation of life-saving therapies yet lack the confidence in their ability to sustain such measures over a period of time, rendering their initial endeavours counterproductive. The authors go on to probe the use of technology to bridge the gaps in care from the level of medical officer to specialist, not only for future pandemic preparedness but for the daily epidemics that continue to scourge overwhelmed public health care services.

### What is known about this topic

- Critical care is not taught as a formal module in undergraduate medical degrees in South Africa;
- Critical care is not offered as a rotation in the compulsory years of internship and community service in South Africa;
- Every doctor would be needed for the COVID-19 pandemic response.

### What this study adds

- Without generalization to the entire intern and community service medical officer population: this study provides some insights into the self-reported comfort of junior doctors to manage critically ill patients outside the ICU during the COVID-19 pandemic;
- This study reveals that there is an ability of junior doctors to provide counselling to families of ill and dying patients and to healthcare workers;
- This study notes that junior doctors do not feel comfortable with triage of patients for admission to ICU.
